# From Heron of Alexandria to Amazon’s Alexa: a stylized history of AI and its impact on business models, organization and work

**DOI:** 10.1007/s40812-022-00222-4

**Published:** 2022-08-04

**Authors:** Lucrezia Fanti, Dario Guarascio, Massimo Moggi

**Affiliations:** 1 Università Cattolica del Sacro Cuore, Milan, Italy; 2 Sapienza University of Rome, Roma, Italy; 3 Westpole, Rome, Italy

**Keywords:** Artificial intelligence, Industrial dynamics, Work organization, O1, 014, O15

## Abstract

This paper explores the development of Artificial Intelligence (AI) and its impact on business models, organization and work. First, we provide a *stylized history* of AI highlighting the technological, organizational and market-related factors fostering its diffusion and transformative potential. We show how AI evolved from being a scientific field to a mostly corporate-dominated field characterized by strong concentration of technological and economic power. Second, we analyze the consequences of AI adoption for business models, organization and work. Our discussion contributes to show how the development and diffusion of this technological domain gives new strength to the lean-production paradigm - in both manufacturing and service sectors - by contributing to the establishment of the new ‘digital Taylorism’.

## Introduction

During the last few decades, the diffusion of smart devices able to ‘learn’ and - thanks to this learning - adapt to changing environments has dramatically transformed the functioning of capitalistic systems. Machine (artificial) intelligence is based on a combination of different advanced technologies capable of reproducing and/or enhancing different human tasks and cognitive capabilities, such as planning, learning or speech and image recognition (Brynjolfsson and Mitchell, 2017; Teddy, 2018; Martínez-Plumed et al., 2020). More precisely, we can define Artificial Intelligence (AI) as a technological domain whose core components are knowledge and techniques aimed at developing *learning systems*, that is: *machines capable of performing specific tasks by fully or partially substituting the human agents and of adapting to changing environments* (WIPO, 2019).^1^

For many decades AI remained a purely scientific domain. Its core knowledge components were developed by universities and research laboratories following the evolutionary trajectory of their relevant scientific and academic fields (e.g., mathematics, physics, engineering, computer science, psychology, neuroscience) and mostly pursuing non-commercial aims (Nilsson, 2010). Then, as the global diffusion of ICTs and the ‘commercialization of the Internet’ take place (Greenstein, 2001; Bonaccorsi & Moggi, 2020), AI becomes a key tool for data-intensive corporate strategies aimed at increasing power, control and profit margins, both within and outside the firm (Rikap & Lundvall, 2021). First, the diffusion of AI pushes forward the automation, robotization (Lane and Saint-Martin, 2021) or, more broadly, the *lean production* frontier.^2^ As productions become more digitized, AI magnifies the effectiveness of monitoring activities providing greater flexibility to fragment processes, reduce bottlenecks and raise productivity. This may increase the labor-saving potential of technological change - in both manufacturing and service sectors - ^−^ by reshaping jobs and labor markets, with a non-neutral impact on income distribution (Gregory et al. 2016; Acemoglu, 2021; Lane and Saint-Martin, 2021). Moreover, AI lies at the core of nowadays dominant business models based on the management of large virtual-physical platforms (e.g., Alphabet, Amazon, Alibaba) relying on learning systems and continuous information processing. For such digital giants, AI is a pivotal component of their complex techno-organizational structure. It allows to tighten control in all directions - i.e. workers, customers, suppliers, complementors and competitors - maximizing rents and value extraction (Dosi & Virgillito, 2019; Coveri et al., 2021; Rikap & Lundvall, 2021).^3^

For what concerns market dynamics, the spreading of AI has been accompanied by a fast concentration process (Shoham et al. 2018; UNCTAD, 2021). On the one hand, the demand for AI goods and services increases across economies and sectors. On the other, such technologies are owned by few corporations capturing most of the profits.^4^ Such a power asymmetry between demand (AI-users) and supply (AI-developers/vendors) is particularly clear in the case of web-services and AI-empowered production, logistics and retail platforms. While digitizing processes and accessing digital markets is vital for the large majority of firms, companies controlling AI technologies tend to operate in oligopolistic or monopolistic conditions (e.g. Amazon Web Services). Such dominant positions have first and foremost to do with: privatization of knowledge (Rikap & Harari-Kermadec, 2020), control of innovation networks (Rikap, 2022), *network effects* (Parker & Van Alstyne, 2018), and strategic planning exerted beyond the company’s formal perimeter (Coveri et al., 2021). In this context, corporations leading the AI technological domain tend to consolidate their position by extracting most of the value from their innovation network (Rikap, 2022).

Indeed, the socio-economic and political impact of AI is increasingly attracting attention in both the academic and policy debate (Furman, 2019; Acemoglu, 2021). On this ground, a growing strand of literature is currently focusing on how AI may impact jobs and income distribution by pointing to the risk of a new wave of technological unemployment (Autor, 2015; Brynjolfsson and McAfee, 2016; Frey and Osborne, 2017; IFR, 2017; Montobbio et al., 2020; Webb, 2020; Lane and Saint-Martin, 2021). On the other hand, more recent contributions have been focusing on the impact of AI-based ‘algorithmic management’ on work organization, job quality and industrial relations (Adam-Prassl, 2019, 2022). Moreover, AI is also at center of those analyses investigating the raise of large digital platforms, the role of ‘data commodification’ and the related concentration of technological, economic and political power (Zuboff, 2019; Gawer, 2021).

This paper adds to the extant literature on AI in different respects. First, the long-term trajectory of this technology is analyzed adopting an evolutionary (Nelson and Winter, 1977, 1982, 2002) and *history-friendly* (Malerba et al. 2016) perspective. Following the theoretical guidance provided by scholars like Freeman, Dosi and Perez (Dosi, 1982, 1988; Freeman & Perez 1988; Perez, 2009), we propose a ‘*stylized history*’ of AI highlighting its shift from a purely scientific to a corporate domain characterized by a strong concentration of technological and economic power. Along these lines, we focus on the interaction between technological, institutional and market-related elements contributing to the diffusion and qualitative developments of AI. *Discontinuities* as well as mechanisms reinforcing *pre-existing capitalistic trends* (Dosi & Virgillito, 2019) that play a crucial role in fostering the diffusion of AI are identified. Second, we analyze the impact of AI on business model, organization and work. By reviewing the most recent literature (Acemoglu, 2021 and Adam-Prassl, 2022, among others), we summarize the major consequences that AI is expected to have in terms of technological unemployment, job quality and labor market fragmentation. Finally, technological developments and market dynamics are empirically documented by providing a comprehensive descriptive analysis. By distinguishing between AI producers and users, we report the evolution of investments and market shares displaying an increasing concentration as well as significant heterogeneities in terms of applications. For what concerns the technological developments, we rely on WIPO (2019) to identify those fields that are directly related to AI components. In line with Martinelli et al., (2019), we analyze AI patents focusing on the direction of technological efforts, the interaction among technological domains and the process of technological concentration/fragmentation triggered by the diffusion of AI.

The paper is structured as follows. In Section 2 we explore the long-term evolution of AI tracing back its stylized history and highlighting the major techno-economic discontinuities. Section 3 analyzes the impact of AI on organization and work focusing on its implications in terms of employment and working conditions. Section 3 provides a descriptive analysis of AI diffusion focusing on patents and market-related data. Section 4 concludes summing up the key results.

## The long-term evolution of AI

The evolution of capitalism as a mode of production and accumulation has been punctuated by the emergence of *General-Purpose Technologies* (GPTs): first, steam power, then electricity and ultimately the development of Information and Communication Technologies (ICTs). The introduction of GPTs represents a structural break (Bresnahan and Trajtenberg 1995; Helpman 1998; Teece 2018). Consolidated economic, social, institutional and cultural configurations start to wane, opening the way for the emergence of new power structures, new markets, new needs and products, and new technologies. Each break coincides with the emergence of a new *techno-economic paradigm* (Freeman & Perez, 1988; Perez, 2009; Dosi & Virgillito 2019), whose advent can be described using Freeman (1991 p. 224)’s words, ‘*the concern here is with the complementarities and externalities of families of interrelated technical, organizational and social innovations…and with the rigidities of the built environment, institutional environment and established technological system*’. Does AI represent a new GPT?^5^. Is it contributing to the so-called ‘Fourth Industrial Revolution’, or should, rather, be interpreted as the long tail of the previous ICT *techno-economic paradigm*?^6^

Providing a final answer to these questions is beyond the scope of our paper.^7^ However, the evolutionary theory provides an helpful guidance to identify key factors shaping the evolution of AI; as well as to assess its impact on the economy. In what follows, we investigate the *historical* evolution of AI (Table 1 in the Appendix summarizes the stylized history of AI) focusing on key factors driving its adoption and diffusion (Malerba et al. 1996a, 1996b, 1997; Zollo and Winter, 2002; Dosi & Nelson 2010) along specific *technological trajectories* (Dosi, 1982; Nelson and Winter, 1977, 1982, 2002; Dosi & Nelson 2016). We build upon the well-established ‘*history-friendly*’ tradition (Malerba et al., 1999; Garavaglia, 2010; Malerba et al., 2016; Capone et al., 2019) carrying out an ‘*appreciative*’ exploration of AI. The aim is to shed light on the convergence of different trajectories as well as on the interaction of supply, demand and institutional factors shaping the evolution of such technology.

We identify three different trajectories whose interaction has enabled the establishment of AI as a technological domain: (i) developments in statistical and computational theory and specific algorithmic techniques; (ii) data availability, strictly linked to the diffusion of the Internet (Cernobbio e Moggi, 2020) and of the exponential growth of connected devices (Unctad, 2021); (iii) improvements in computational power and data storage capacities. These trajectories are punctuated by specific advancements in terms of knowledge, techniques and applications, e.g. *Machine Learning*^8^ (ML) and *Artificial Neural Networks*^9^ (ANNs) *tools*. The diffusion of AI and the growing variety of its applications is also related to the advent of data intensive business models as well as to the increasing fragmentation of economic and production relationships. In this respect, AI-based smart machines and adaptive learning systems make it possible to efficiently manage large and geographically dispersed networks, e.g., communication, production, financial, logistics and retail networks. By the same token, the ubiquity of the Internet across industries, markets and societal domains favors the flourishing of business models and product innovations based on ‘data-hungry’ AI technologies.

### The advent of intelligent machines

For about one century, AI remained confined to a purely scientific-academic sphere with few or none industrial and business applications. Scientific efforts were largely driven by an ancient ambition, dating back at least to Leonardo da Vinci, that is to create autonomous, intelligent machines, able to do what man would like to but cannot do (e.g., flying, predicting the future, solving ‘impossible’ mathematical problems). Things start changing between the 1980s and the 1990s, with the diffusion of microprocessors, digital devices, connected computers and then the establishment of the Internet (Greenstein, 2001; Bonaccorsi & Moggi, 2020; O’Mara, 2020). From that moment on, AI starts attracting increasing attention and growing amounts of capital from the corporate world.^10^ Quickly, AI becomes crucial for the newborn web-based business models relying on data extraction^11^ and processes’ automation. For companies controlling large amount of data (e.g., large digital platforms as Alphabet, Alibaba or Amazon), the opportunities to develop new applications and profit from AI grow exponentially.^12^ On the other hand, AI-related innovation (and knowledge) becomes increasingly privatized and protected (see WIPO (2019), Unctad (2021), Rikap & Lundvall (2021) and Sect. 4 for an analysis of patenting in the AI domain), reversing the original trend that had characterized the early academic-based stages of AI. In this context, large corporations relying on data-intensive business models become also AI developers, patentees and vendors, selling to adopters widespread all across sectors and countries.

*A stylized history of AI*

The ancient origins of AI, broadly understood as the attempt to ‘automatize’ human activities, can be dated back to centuries, if not millennia. One of the first attempts is that of Heron of Alexandria, in the 1st century BC (Bedini, 1964). In Heron’s numerous surviving writings, there are designs for ‘automata’, i.e. machines operated by mechanical or pneumatic means. Heron’s rudimental automata were aimed at instilling ‘faith by deceiving believers’ displaying ‘magical acts of the gods’ as those pursued by a statue that poured wine. Likewise, the logical machine invented by R. Lullo drawing on his “*Ars Magna*” (1308) represents the first attempt to realize a mechanical calculator able to reproduce human computation abilities (Fidora & Sierra, 2011). Lullo’s efforts, in turn, inspired Leibniz’s “*Dissertatio De Arte Combinatoria*” (1666), one of the ancestors of modern ‘computational thinking’ whose echo can be identified in the ‘analytical engine’ project developed^13^ by C. Babbage (1837).

Between the 19th and the early 20th century, major achievements in statistical and probabilistic theory are reached: from Legendre’s Least Square method (1805), thereafter widely used for data fitting problems, to Bayes’ studies and the formalization of the ‘Bayes theorem’ proposed by Laplace (1802), to the introduction of the ‘Markov chains’ (1913). These are the key theoretical foundation (mostly related to probability and analysis of stochastic processes) upon which the subsequent efforts to realize AI will move. Building on such theoretical grounds the first crucial steps towards modern informatics and AI as we know it are made. Alan Turing contributions range from algorithmic and computational theory - as his famous ‘*Turing machine*’ representing the direct ancestor of modern computers^14^ - to logical thought and investigation into machine ‘*intelligence*’ (the well-known *Turing test*). After some innovative advances in the field of ANNs between the 1940s and 1950s, such as the Threshold Logic Unit (TLU) in 1943 and the first neural network machine, i.e. the Stochastic Neural Analog Reinforcement Calculator (SNARC) in 1951, another major breakthrough came in 1956, during a seminal workshop organized by J. McCarthy and other computer scientists including the future Nobel Prize H. Simon, at the Dartmouth College in New Hampshire.

Following the Dartmouth discussions, Newell and Simon created a program, *Logic Theorist* (LT), capable of imitating some kind of ‘reasoning’ by proving theorems starting from mathematical principles. LT is the first algorithmic attempt to imitate human heuristics and cognitive processes. In 1958, F. Rosenblatt, working at the Cornell Aeronautical Laboratory, introduced the ‘perceptron’. The latter is an ML algorithm, originally implemented as an ANN machine for image recognition (the ‘Mark I perceptron’), causing a considerable stir among the US media and, for the first time, attracting a widespread attention over AI.

Despite during the 1960s probabilistic theory was extensively applied to advances and developments of ML algorithms, the 1970s have been defined as the ‘AI winter’. This is due to a slowdown in AI-related research projects and an overall slowdown of AI research.^15^ On the other hand, the multidisciplinary AI scientific community was working hard to agree on a univocal definition of it. In his book “*The Science of the Artificial*” (1996) H. Simon wrote **«***The phrase “artificial intelligence”, […] was coined, I think, right on the Charles River, at MIT. Our own research group […] have preferred phrases like “complex information processing” and “simulation of cognitive processing”. But then we run into new terminological difficulties […]. At any rate, “artificial intelligence” seems to be here to stay.***»**. This passage provides an historical account of the divide between what AI really was (and is) and what computer scientists wanted it to be (i.e. the soul of a ‘truly’ intelligent machine). Simon (1985, 1996) have shown how the human rationality is limited by the amount of information that our brain can simultaneously process. However, humans ‘have’ something that is really hard to reproduce with a machine: their ability to learn by using and adjusting previous information to solve unknown (and unexpected) problems. In the 1980s, the AI winter comes to an end. Research on ANNs saw a new momentum thanks to some major advances, including: the pioneering work of K. Fukushita on the ‘neocognitron’ (1979), the implementation of Recurrent Neural Network (RNN) models (1982), backpropagation techniques to train ANNs (1986) and the Convolutional Neural Networks^16^ (CNNs) intensively applied to image recognition and classification.

In this phase, thanks to renewed interest in ANNs and the availability of new human-machine interfaces, AI systems started to be adopted by large US companies, such as Digital Equipment and DuPont. Later on, *expert systems* programs, the ancestors of the latest *intelligent systems*, started spreading among large companies, mostly located in the US, Japan and the UK. This was the beginning of the AI industry. In this period, the diffusion of AI was also facilitated by the development of *General Purpose Machine Learning*^17^ (GPML) (Taddy, 2019) opening the way to business activities such as ‘Data Mining’ and ‘Predictive Analytics’.

Between the 1990s and the early 2000s, AI applications based on ML starts growing exponentially marking a turning point between a mostly theoretical research domain to a more applied IT solution. In 1995, the first work on Support Vector Machines (SVM) is presented by C. Cortes and V. Vapnik. This is a pivotal tool to solve Natural Language Processing (NLP) problems, opening the way for the flourishing of research in this field. The latter is of course also driven by the increasing availability of data and digitized information linked to the Internet. Pivotal is also the work by S. Hochreiter and J. Schmidhuber who introduced, in 1997, the Long Short-Term Memory Recurrent Neural Network^18^ (LSTM-RNN) architecture, from which the subsequent developments in the field of DL algorithm will follow. At this stage, the main research efforts came from few big players operating in the computer industry. The companies investing more in AI were IBM, Hitachi and Toshiba (Martinelli et al., 2019). In terms of market dynamics, a constellation of new entrant start-ups was rising, especially in the US and the UK. At the same time, future US Big Tech oligopolists – e.g., Alphabet and Amazon – were taking their first steps. Indeed, the peculiar nature of AI favored the development of start-ups. In many cases, their successful entry is to a large extent explained by the ideas of a few brilliant programmers and scientists. However, the technological and economic advantage enjoyed by large players make most of these start-ups too easy preys. Significant examples are those of Deep Mind (a UK start-up producing frontier ML solutions) acquired by Alphabet in 2014 or Maluuba, a Canadian AI developer start-up, acquired by Microsoft in 2017.

The early 2000s are indeed a crucial moment. From being mostly computational machines (programs) aimed at solving high-dimensional but still finite combinatorial problems, such as IMB’s *Deep Blue* (1997), AI assumes the form of learning systems capable to replicate things that are more and more similar to complex reasoning, as in the case of IBM’s *Watson* (2011) (Dosi & Virgillito, 2019). From a technological point of view, AI grows rapidly thanks to huge improvements in computational and processing capacities of related technologies, mainly ML algorithms using DNNs. Thanks to AI, ‘old computational problems’ can now be solved in a very short time, with relevant implications for both scientific advancements and industrial innovation (Cetrulo and Nuvolari 2019). In addition, AI-based machines and computer systems capable to act ‘as humans’ begin to emerge, e.g., recognizing images, sounds or texts without prior instructions; solving unexpected problems; and accomplishing tasks even in continuously changing contexts (for a detailed account of major AI-based innovations introduced in this period, see, among others, Brynjolfsson and McAfee, 2014; Quintarelli, 2019). As mentioned before, these developments are partly related to the expansion of a ‘virgin land’ for capitalist accumulation, i.e. the Internet, providing inexhaustible amounts of data (and opportunities) to feed AI research and applications (Greenstein, 2001; Coveri et al., 2021; Rikap & Lundvall, 2021).^19^ Indeed, it is already since the 1980s that private corporations starts colonizing AI (O’Mara, 2020). Focusing on private-public R&D collaboration, Zhang et al., (2021) show that industry-university research centers and agreements tend to increase steadily over time.^20^ Reporting data on academic-corporate peer-reviewed articles published during the 2015–2019 period, they highlight that the United States produced the highest number of academic-corporate AI publications—more than double the amount in the European Union, which comes in second, followed by China in third place.^21^ An even more thorough account of such transition into the corporate domain is provided by Rikap & Lundvall (2021). These authors focus on the role played by ‘Big Tech’ in the AI domain, showing how key American and Chinese players - i.e. Alibaba, Alphabet, Amazon, Microsoft, Tencent – take the lion share concerning both patents (see Sect. 4) as well as AI jobs. Regarding applications, a large chunk of investments on AI has been directed towards private Internet-related activities such as: search machines, gaming, social media and e-commerce.^22^ No less relevantly, key AI technologies as those related to speech and image recognition are attracting significant public investment related to the defense and security sectors.

From an historical perspective, the spread of AI can be represented as a key turning point in the long-lasting, complex interplay between humans and machines, and between *natural* and *artificial* worlds (Simon, 1996). According to our stylized history, AI can be described as the cumulation of *incremental innovations* combining through the convergence of different technological trajectories (Dosi & Nelson, 2016) within the broader ICT *techno-economic paradigm*. Following Perez (2009), the evolution of AI within the ICT techno-economic paradigm can be framed as follows: semiconductors as motive branch; computer, software and smartphone producers as carrier branch; and Internet as the main infrastructure.

As argued, the diffusion of AI has not followed a linear pattern. In its early stages, AI represented an academic niche, with few industrial and business applications. Its development, was mostly fueled by scientific research (involving various research fields and benefiting from international cooperation amongst researchers) aiming at realizing something expected to be ‘as close to human intelligence as possible’. At the dawn of the new millennium, the interplay of *technological*, *market* and *institutional* factors paved the way for a ‘great leap’, which took the form of a *discontinuity* along the pattern of adoption and diffusion of AI systems. Starting in the ICT and high-tech sectors, AI technologies rapidly became ubiquitous. Soon after, embodied in artefacts that are at the heart of most production activities (e.g., electronic payment systems, customer-care services, ID-recognition services, etc.), AI starts to penetrate all the economy’s interstices. As a result, while a dominant share of industrial investments is concentrated in few countries, sectors and dominated by large corporations (Lee et al., 2018; Martinelli et al., 2019; Rikap & Lundvall, 2021) AI becomes a key component of consumers’, producers’ and public operators’ everyday lives.

*Techno-economic discontinuities*

In what follows, the stylized history of AI is complemented by a systematic account of the key technological discontinuities contributing to accelerate its diffusion across industries and countries. The availability of large amounts of digitized information is one of the key elements. In fact, data represents the ‘nourishment’ of machine intelligence. The more digitized information is available, the greater will be the opportunity for machines to learn and become ‘smarter’. From an infrastructural point of view, the rapid diffusion of networks enabling widespread connectivity increases the opportunities for data generation, storage and transmission. Similarly, the massive diffusion of connected objects (e.g., smartphones, cars, houses, machineries) multiply the ‘events’ that can be transformed into inputs for AI learning systems. A crucial role is played by developments in the field of semi-conductors and super-conductors. Starting in the 1980s with the introduction the Complementary Metal-Oxide-Semiconductors (CMOS) technology, incremental innovations in this field went hand in hand with expanded opportunities for AI’s diffusion.

With the establishment of the Internet (Greenstein, 2001; Cernobbio and Moggi, 2020; Bonaccorsi & Moggi 2020) the amount of data to be used for AI development grows further. In this context, a number of tools allowing to process data in a non-linear and not exclusively deterministic way are introduced. This magnifies AI technologies learning and predictive capabilities. On the other hand, increasing size and variety of data means enlarging the areas open to AI’s applications (e.g., natural language processing, sound, image and pattern recognition, speech-to-text conversion, etc.). By the same token, innovations such as *ML algorithms, Reinforcement Learning* (RL) mechanisms, *In-Memory Databases*^23^ (IMDB), Not only-Structured Query Language^24^ (NoSQL) as well as the ‘Spark Apache’ open source framework increase AI’s ability to learn from Big Data. All these instruments are specifically designed to manage large and unstructured dataset reducing both physical and computational costs.

Some of the most popular ML applications are related to gaming. Among the others, there are Google’s *AlphaGo* (2016), an AI system implemented to play the ancient Chinese game Go, or the highly modular AI system implemented by Maluuba (Microsoft) to play Atari’s Pacman game (2017). The latter, by adopting RL instead of *Supervised Learning* (SL) mechanisms, is able to break down the entire game into different tasks each of which is performed by a parallel DNN routine within a *Hybrid Reward Architecture*^25^ (HRA).

However, *game*-*solver* ML algorithms are based on strict rules requiring codified knowledge, whereas ML algorithms applied to *real-world* interactions require both codified as well as specific knowledge (i.e. theories governing the specific knowledge domain wherein they are expected to operate). Most of the research oriented to industrial AI focuses on learning systems relying on both codified and uncodified knowledge. This is mostly pursued by the combination of ML DNNs tools with other complementary innovations (Teddy, 2018; Martinelli et al., 2019).

Key to the growth and development of AI are also improvements in the field of data storage. To maximize the learning benefits stemming from unstructured data, companies need to move away from data silos or data lake storage models. To improve their performance, AI technologies need massive, highly scalable and parallel data hubs. Cloud architecture and all-flash storage solutions are designed for this purpose (infrastructure designed not to simply store data but to share it). Since the 2000s, diffusion and improvements of cloud storage provide further boost to AI. On the other hand, corporations dominating cloud technologies and infrastructures (e.g., Amazon, Microsoft) gain a significant comparative advantage becoming technological leaders and increasing the amount of value extracted from AI (Coveri et al., 2021). As pointed out by Rikap & Lundvall (2021 p. 85), dominating cloud infrastructures allows key corporations (e.g., Amazon Web Services) to subordinate competitors to their own strategies. A paradigmatic example is that of Netflix that admitted her total dependence on AWS to carry out her activities. Focusing on this matter and carrying out an extensive analysis of patent data, Martinelli et al., (2019) stress the importance of technological improvements in areas such as *low energy consumption sensors* and *cloud connectivity* tools.

## The impact of AI on business model, organization and work

As pointed out by Perez (2009), techno-economic paradigms are characterized by the change or reinforcement of peculiar organizational practices. Organizational and technological change are in fact two sides of the same coin. This is the case, for example, when we look at the Tayloristic workplace organization, becoming dominant during the Fordist mass production era (Braverman, 1974) and being the indispensable counterpart of efficiency-enhancing and labor-saving process innovations. Within the Tayloristic paradigm, setting a specific organizational framework - i.e. fragmentation, standardization and codification of work tasks - becomes an unavoidable pre-condition for technological change and innovation. In turn, the diffusion of new technologies is likely to facilitate further organizational innovations. From an evolutionary perspective, the adoption of new technologies also depends on firms’ idiosyncratic dynamic (organizational) capabilities (Zollo and Winter, 2002; Dosi et al. 2010). The latter are heterogeneously distributed among firms, reflecting their specificities in terms of knowledge base, behavioral patterns, routines and hierarchical arrangements (Dosi and Marengo, 2015).

The interplay between companies’ economic aims - e.g. increasing efficiency and reducing costs - and organizational innovations is also relevant to explain the diffusion of AI technologies. Within manufacturing sectors, the introduction of smart machines able to ‘learn’ and recognize images and sounds allows to strengthen the ‘lean production’ organizational trajectory (Coriat, 1991a, b; Musso, 2013; Cirillo et al., 2021). Thanks to AI, efficiency gains reach unimaginable levels as compared to the beginning of the lean techno-organizational trajectory back to the 1970s. A number of channels are in operation, such as: (i) reduction of the amount of labor input used in production; (ii) maximization of both human and machines’ efficiency in performing their tasks; (iii) new opportunities for machine-assisted human activities; (iv) fixing of bottlenecks and information feedbacks that allows continuous quality improvement. Outside the plant, AI technologies provide powerful means to control supply chains and interact with competitors, suppliers and customers. By facilitating the time-space fragmentation and monitoring of tasks - regardless of where these tasks are carried out - AI deepens the process of flexibilization and externalization of production, both in manufacturing and services.

One of the side-effects is the entry of the lean techno-organizational logic into the service sector (Dosi & Virgillito, 2019). A paradigmatic example relates to labor platforms (Bogliacino et al., 2019; Vallas & Schor, 2020). Relying on AI technologies, these platforms control workers and service providers even if they are located miles apart. At the same time, ML algorithms are used to control and improve workers’ performance by giving awards and imposing penalties. Focusing on platforms, Dosi & Virgillito (2019) describe the combination of AI technologies and the lean organizational set-up as a brand new ‘Digital Taylorism’.

In this context, the tendency towards an increasing fragmentation of production is likely to stimulate the design, adoption and use of AI tools capable of reproducing *routine* tasks (blue collar) based on highly codified knowledge and specific rules. This may have a significant impact on labor markets with the destruction of jobs, in both manufacturing and services, and a polarizing effect on income distribution (Acemoglu, 2021). A disruption that could be magnified by AI’s capacity to interpret ‘unstructured’ data, that is data referring to complex environments as those faced by humans. As Acemoglu (2021) emphasizes, thanks to learning systems based on unstructured data, AI may learn how to replicate *non-routine* complex tasks (i.e. white-collar tasks based on non-codified rules, experience and complex knowledge).

Martínez-Plumed et al., (2020) recently proposed a theoretical approach to map AI technology benchmarks, labor tasks and cognitive abilities. This approach provides a fine-grained mapping of the human cognitive abilities that AI may reproduce, enhance or substitute. In particular, they show how jobs traditionally considered as non-substitutable given the significant amount of cognitive abilities they entail, are now actually threatened by AI.^26^ From an empirical standpoint, these arguments seem to be confirmed by Montobbio et al., (2020). On the one hand, they show that a large amount of AI patents is concentrated in human-intensive industries, such as logistics or health and medical activities. On the other, by carrying out a textual analysis of AI patents they document the growing relevance of ‘labor-saving heuristics’ associated with the usage of machines and robots.

Alongside the risk of job destruction due to automation and the adoption of lean-production arrangements, there is an army of fragmented, often unqualified and mostly low-payed workers growing *behind* the ‘intelligent’ machines (Vallas & Schor, 2020; Coveri et al., 2021). A significant example is Amazon Mechanical Turk (AMT).^27^ This is a crowdsourcing Internet service providing microtasks (*Human Intelligence Tasks*, HITs) performed on-demand by human workers (the so-called ‘*turkers*’)^28^ (Irani and Silberman, 2013) that are directed by a global AI-based digital platform. AMT workers are geographically dispersed, pervasively monitored, and subject to high levels of exploitation. More specifically, Amazon’s AI technologies and cloud services ensure the matching between supply and demand, monitoring and maintaining productivity levels.^29^ Indeed, empowering commonly used products – such as cars (Tubaro & Casilli, 2019) – with AI technologies may further increases the demand for (digital) unqualified labor. These workers are asked to ‘support, maintain and train’ the ML algorithms by empowering AI systems. Focusing on AI-based platforms linked to the French automotive sector, Tubaro & Casilli (2019) document how smart car assistants’ operations are strictly connected to a large mass of platform workers largely located in French-speaking African countries. Their aim is to train French-speaking car assistants so to ensure a continuous improvement of their performance (e.g. helping them to disambiguate or recognize new words).

When we look at the labor impact of AI, a peculiar dualism seems thus to emerge: on the one hand, as we previously discussed, this technology may destroy jobs down the assembly line. On the other, it boosts the demand for micro-tasks performed by spatially dispersed (and highly exploited) platform-micro workers (De Stefano, 2016). A similar army of micro-workers is activated to support the operations of AI ML-based technologies supporting large internet platforms (e.g. Alphabet’s Google). Every day, thousands of workers are employed to feed, clean, fix and train algorithms and learning systems. Among the more relevant tasks, there are some ‘problem solving’ activities that AIs alone cannot perform, such as deciding on the ethical or political suitability of a web content. At the top, the highest profiles in fields such as mathematics, physics and computer science are attracted by the R&D headquarters of digital corporations (Vallas & Schor, 2020).

Finally, the adoption of AI systems is also reshaping organizational patterns and the way large corporation interact with workers, customers, suppliers and competitors. A clear example is Amazon. AI technologies allow to combine lean production logic at the warehouse level, Big Data processing to ‘anticipate’ demand patterns, and ML-based systems to maintain tight control of the supply network. In this way, both internal and external efficiency are maximized. Moreover, for what concerns innovation, corporations like Amazon – and the same goes for the Chinese Alibaba and Tencent - exploit their comparative advantage to dominate the data-intensive AI’s technological trajectory taking over innovative start-ups (Rikap & Lundvall, 2021; Coveri et al., 2021) and introducing new products aiming at lock-in both customers and suppliers. A case in point is the Amazon’s virtual assistant Alexa. The latter is the outcome of an acquisition^30^ that took place in 2013 and is built on a ML technology, i.e. Amazon Echo. Once installed, Alexa turns the home into an extension of the Amazon marketplace, by exponentially increasing the likelihood of new purchases. On the other hand, Alexa is the Amazon’s eye that syphons out all data flowing inside the house. In so doing, Alexa learns and becomes more efficient. Amazon, on the other hand, increases her technological advantage and its broad socio-economic power. Furthermore, for those corporations like Amazon, AI is also crucial to implement personalized advertisement strategies - as in the case of the *Amazon recommendation engine* - or even to propose an AI-empowered fully ‘automatized’ shopping experience at the Amazon *Go Stores* (Kenney et al., 2021).

## The recent development of AI: a descriptive analysis

In this Section, the diffusion of AI technologies is analyzed by focusing on: (i) investments by type of technology; (ii) market dynamics and applications; (iii) start-up demographic patterns and acquisitions; (iv) patents.

As we will see, the diffusion of AI technologies is actually reinforcing the overall trend towards market concentration characterizing the ICT techno-economic paradigm. Indeed, by exploiting their technological comparative advantage and acquiring most of the more promising start ups, few Big-Tech players are consolidating their dominant positions (Unctad, 2019; WIPO, 2019). Moreover, we will show how this oligopolistic configuration is also reflected by the distribution of patents, characterized by a small group of companies owning the vast majority of AI-related patents.

Data are collected from Statista^31^, that is a business data platform providing survey-based information on different economic and financial dimensions related to consumers, companies, sectors and market dynamics; whereas AI patent descriptive analysis refers to the WIPO Report (WIPO 2019).

*Investments and diffusion*

**Fig. 1:**
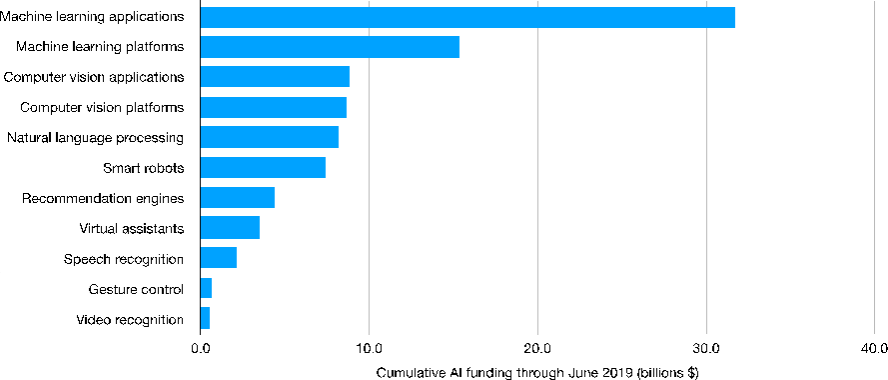
Worldwide AI cumulative funding (2019) by technological category^32^ (billion U.S. dollars) Source: Authors’ elaboration on Statista data

We start our descriptive exploration by focusing on the supply-side of the AI industry, that is on companies developing AI technologies. First of all, we look at the recent trends of AI corporate investments. In this way, we are able to shed a light on the diffusion of AI technologies in terms of both the intensity of investments and their qualitative composition (i.e. type of AI technology). Figure 1 shows the amount of worldwide spending on AI technologies between January and June 2019. As we can see, a large amount of AI-related investments is concentrated in six technological areas. The lion’s share goes to ML applications and platforms, amounting, respectively, to 31.7 and 15.3 billion U.S. dollars. However, a non-negligible share of the overall spending relates to computer vision and platforms as well as to natural language processing (8.8, 8.7 and 8.2 billion of U.S. dollars) and smart robots. A significantly smaller amount of spending characterizes, in turn, domains such as virtual assistants, speech and video recognition and gesture control.

**Fig. 2:**
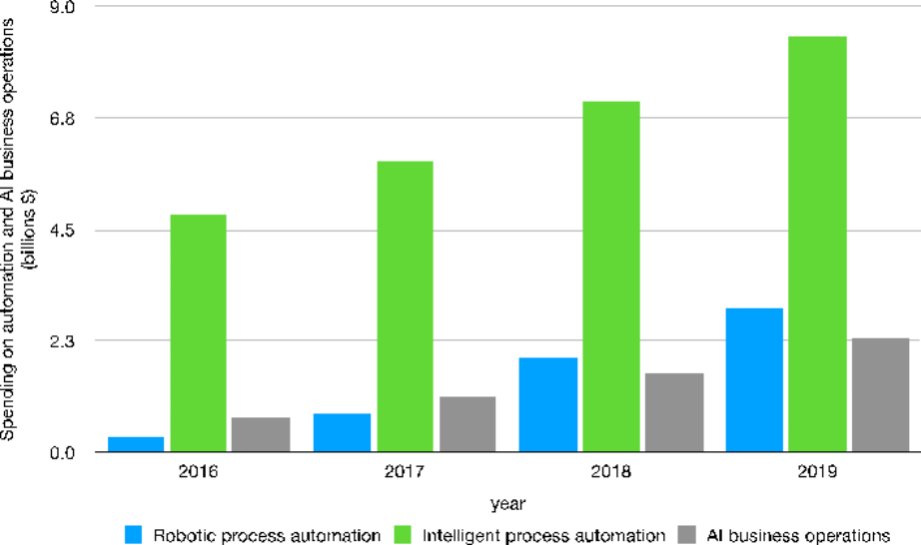
Worldwide spending in automation (RPA/IPA) and AI business operations 33(billions U.S. dollars) – 2016–2019 Source: Authors’ elaboration on Statista data

In terms of dynamics, AI investment displays constant growth (Fig. 2) concerning both robots and Intelligent Process Automation (IPA) as well as AI business operations. Both RPA and IPA show a substantial spending increase over a relatively short time-span (2016–2019). This seems to suggest that the diffusion of AI technologies is, at least in relation to corporate investments, driven to a considerable extent by process innovations aiming at increasing organizational efficiency. By the same token, as discussed in Sect. 3, the significant share of investments directed to RPI points to an intensification in the diffusion of AI-related technologies in manufacturing industries relying on smart robots as a way to reduce costs and increase efficiency.

**Fig. 3:**
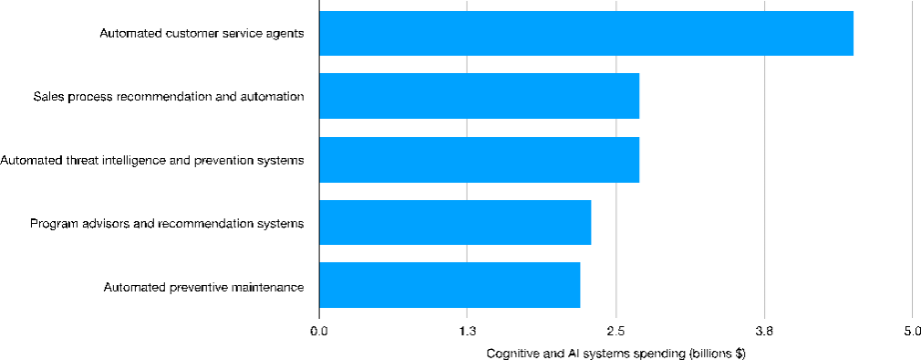
Worldwide cognitive and AI systems spending (billion U.S. dollars) by use case^34^ − 2019 Source: Authors’ elaboration on Statista data

Focusing on services, (automated) customer care is the area attracting the largest amount of investments (more than 4.0 billion dollars) followed by sales process recommendation (2.7 billion) (Fig. 3). In both cases, AI might help to automate cognitive tasks characterized by a medium-high degree of repetitiveness. In this respect, the introduction of AI technologies seems again to aim at reducing the amount of labor input used for production. As emphasized by Dosi & Virgillito (2019), such developments - i.e. the intensive use of digitalization and automation technologies in the service sector - might be interpreted as the transposition of the Tayloristic organizational logic from manufacturing to the service sectors. As for the other use cases, most of the AI-related investments turn out to be concentrated in areas related to security, quality control and maintenance. The increase of security-related investments is linked to the data intensive nature of AI, requiring continuous upgrading in terms of cybersecurity and privacy standards. As for quality control and maintenance, these areas are again linked to process innovations designed to reduce inefficiencies and costs.

**Fig. 4:**
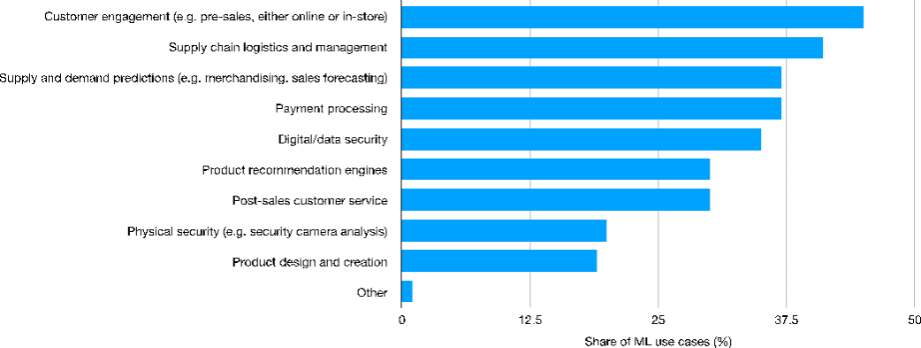
Worldwide Machine Learning use cases in the retail industry^35^ (%) − 2019 Source: Authors’ elaboration on Statista data

Partial confirmation of the Digital Taylorism hypothesis (Dosi & Virgillito, 2019) is provided in Fig. 4, where AI investments are analyzed by focusing on the retail sector’s use cases. In 2019, the largest share of use cases was related to customer engagement (45% of companies exploiting ML adoption for customer engagement). This reflects the huge improvements allowed for by AI technologies in terms of customer engagement, especially during the phases of product design. In this respect, companies rely on AI technologies in order to both tailor products to the customers’ needs and preferences, and to further improve the relative efficiency of processes by continuously adjusting them according to the changing market needs. The second ranked use case is directly related to process efficiency, that is supply chain logistics and management (41% of those companies recording a use case), while the third one is significantly related by involving supply and demand predictions. An important role is also played by payment services, customer care and data security services.

**Fig. 5:**
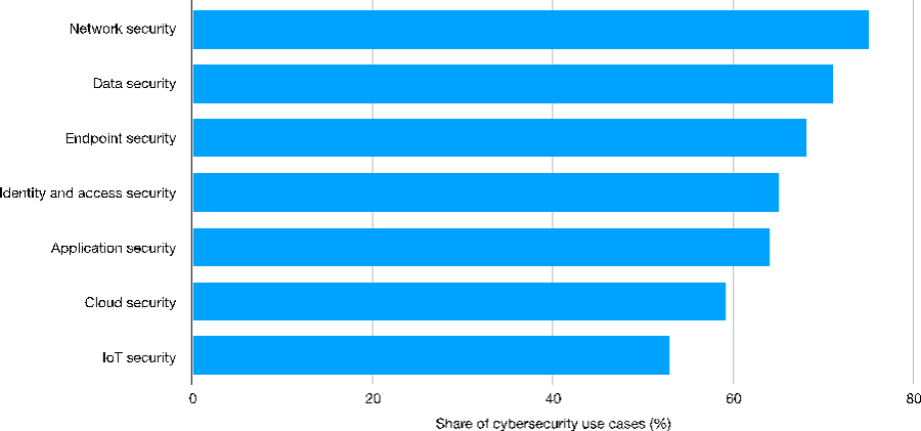
Top cybersecurity use cases in organizations^37^ (%) − 2019 Source: Authors’ elaboration on Statista data

By proceeding with our exploration of the AI diffusion, Fig. 5 shows the distribution of corporate use cases in cyber and data security.^36^ As argued, the use of AI-based technologies requires parallel investments (and organizational efforts) in terms of security. This is attested by the fact that security-related use cases are homogeneously distributed across the AI domains. The areas characterized by the largest number of use cases are network (75%) and data security (71%). With regard to these domains (as well as the others listed in Fig. 5), such a high concentration of use cases could be associated to the fact that almost all AI technologies and devices imply the use of digitized information networks. Therefore, ensuring networks and data protection may represent a pre-condition for safely operating with the AI technologies. On this ground, by focusing on Italian companies Cirillo et al., (2022) have recently documented how the adoption of Industry 4.0 technologies – among which we find either AI and other technologies related to its domain, such as Internet of Things (IoT) and Big Data – is mostly concentrated in Cybersecurity and web applications.

**Fig. 6:**
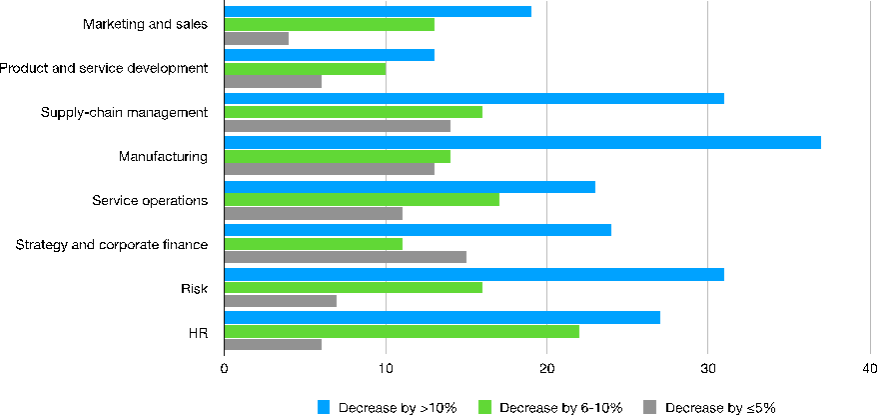
Worldwide cost decreases from adopting AI in organizations by function^38^ − 2019 Source: Authors’ elaboration on Statista data

To conclude our supply-side descriptive analysis, we show the impact that AI adoption may have in terms of cost reduction by discriminating for the type of activity these technologies are adopted for. As we can see from Fig. 6, in almost all the activities affected by the introduction of AI, the expected cost reduction is over 10% for the majority of companies included in the survey.

The most significant reductions are observed in manufacturing (with 37% of companies reporting a decrease in costs over 10%), supply chain management and risk management (31%). A considerable cost reduction is also recorded in service-related activities like human resources (27%), strategy and corporate finance (24%) and marketing (18%). Thus, the efficiency-enhancing effect of AI seems to be confirmed in both traditional manufacturing activities and service-oriented ones.

*Demand-side*

**Fig. 7:**
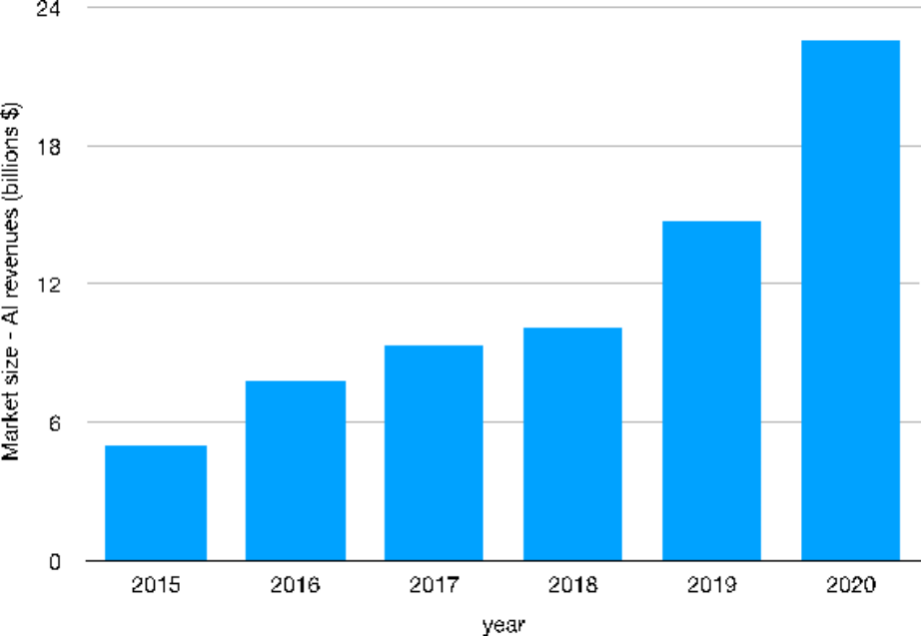
Worldwide AI market size in terms of revenues^39^ (billions U.S. dollars) – 2015–2020 Source: Authors’ elaboration on Statista data

By focusing on users we now empirically explore the demand-side of AI industry. First of all, we look at the time series of market revenues related to AI products and services in order to shed a light on the evolution of the overall AI market in terms of size. As we can see from Fig. 7, in 2020 the AI market reached a dimension four times larger as compared to 2015, by rising from 5 to 22.6 billion U.S. dollars.

**Fig. 8:**
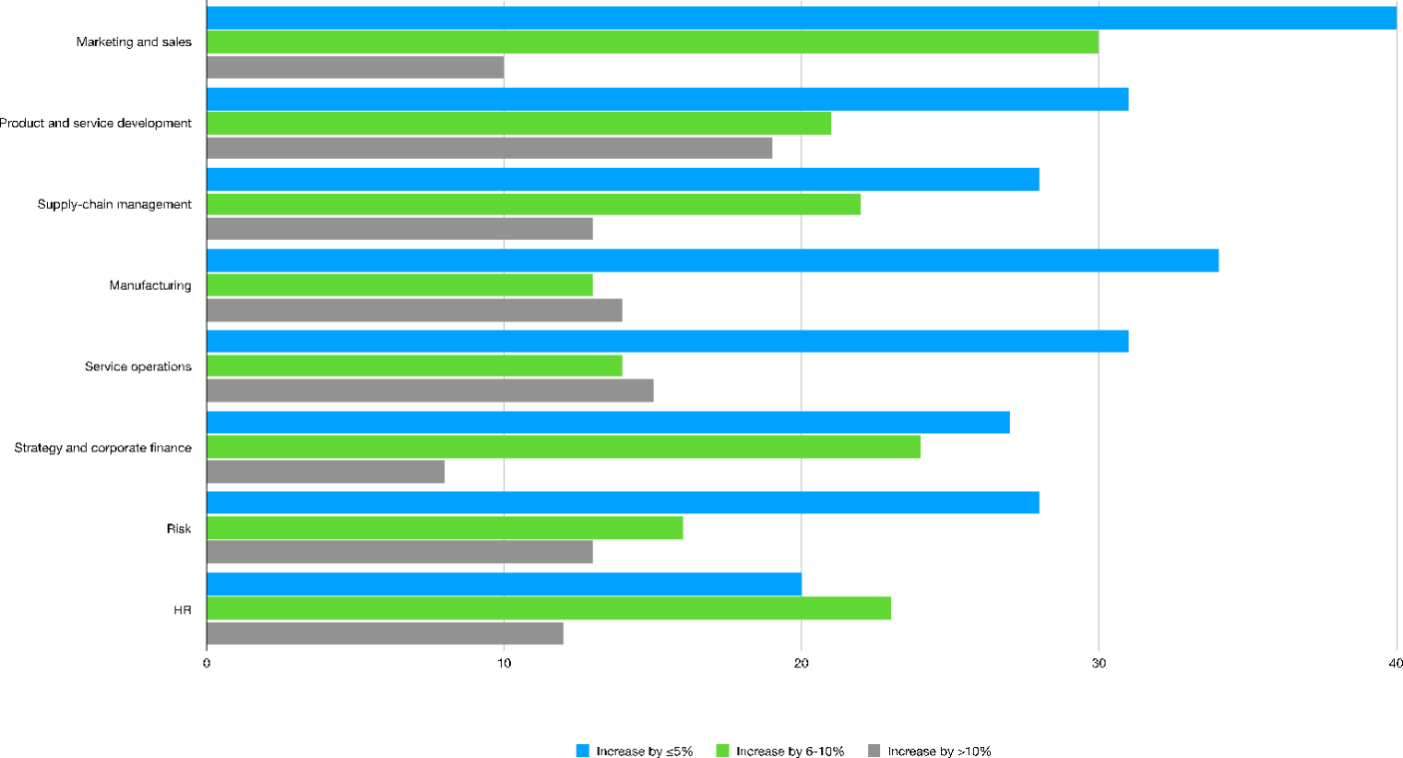
Worldwide revenue increase from adopting AI in organizations by function^40^ − 2019 Source: Authors’ elaboration on Statista data

By reflecting the evidence reported on the decrease in relative costs associated with the use of AI technologies (see Fig. 6), the relative increase in revenues associated with the use of AI in a variety corporate activities now comes under focus. Unlike the previous findings on the cost-reduction and efficiency enhancing effects of AI, Fig. 8 shows that, on average, the majority of adopters reported an increase in revenues of less than 5% (blue bars). This is particularly evident when we look at marketing and sales (40%), manufacturing (34%) and service operations (31%). An increase ranging between 6 and 10% (green bars), is in turn mostly associated with marketing and sales (30%), strategy and corporate finance (24%) and human resources (23%). On the other hand, the higher share of companies increasing their revenues by more than 10% through adoption of AI technologies is related to product and service development (19%), manufacturing (15%) and service operations (14%).

**Fig. 9:**
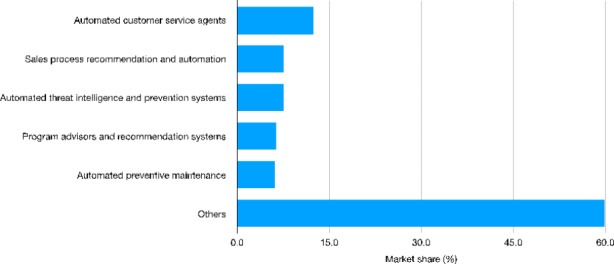
Worldwide top use cases of cognitive and AI systems by market share − 2019 Source: Authors’ elaboration on Statista data

Finally, Fig. 9 ranks AI use cases according to companies’ market shares in 2019. As can be seen, automated customer service agents account for 12.5% of the AI use cases and cognitive systems, followed by sales process recommendation and automation, and automated threat and prevention systems accounting for 7.5 and 7.6%, respectively.

*Market concentration and patent dynamics*.

We now provide some evidence on the structural evolution of AI markets. First of all, we must point out that we are still dealing with a relatively small market (around 10 billion US dollars) as compared to the overall IT (3.8 trillion US dollars) and software (450 billion US dollars) markets. However, given the increasing ‘hype’ around the diffusion of AI technologies and the consequent potential business opportunities and transformations following from its developments, it is crucial to investigate how this market is structured, who the key players are, and how it may evolve in the near future.

Consistently with our stylized-history of AI (see Sect. 2), we now empirically investigate whether the spread of such data-intensive technologies is accompanied by an increasing degree of market concentration. The latter might in fact be generated by the peculiar and intrinsic characteristics of AI technologies. In this domain, technological advances and innovations – especially those related to ML and Big Data - are in fact characterized by a significant degree of *cumulativeness*. Companies having a comparative advantage concerning technologies and competences that are relevant to the development of AI are likely to increase and consolidate their market positions at the expense of existing and potential competitors. At the same time, given their knowledge-intensive and scalable nature, AI technologies leave room for start-ups that by introducing new products may find a gap in the market. Nevertheless, the modular nature of AI technologies can counterbalance such tendency by again penalizing start-ups and favoring concentration. In order to develop their products, start-ups must rely on incumbents’ platforms and services. This obviously increases the probability of acquisitions, leading to further market and technological concentration (Unctad, 2019).

**Fig. 10:**
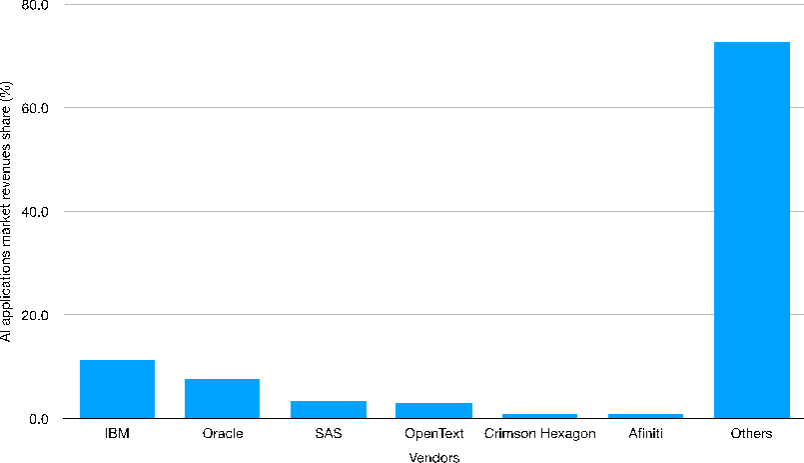
Worldwide AI applications market revenue share by vendor^41^ - in 2018 Source: Authors’ elaboration on Statista data

**Fig. 11:**
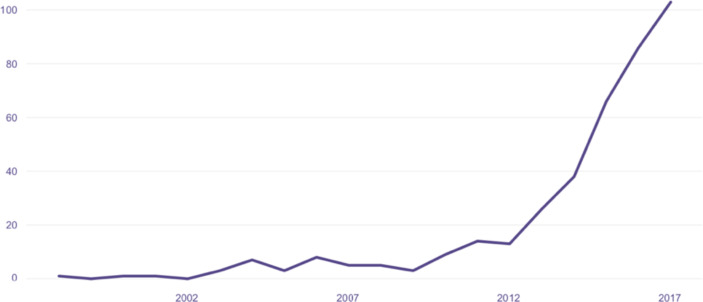
Number of acquisitions in the AI sector by the acquisition year – 1997–2017 Source: WIPO Report 2019

**Fig. 12:**
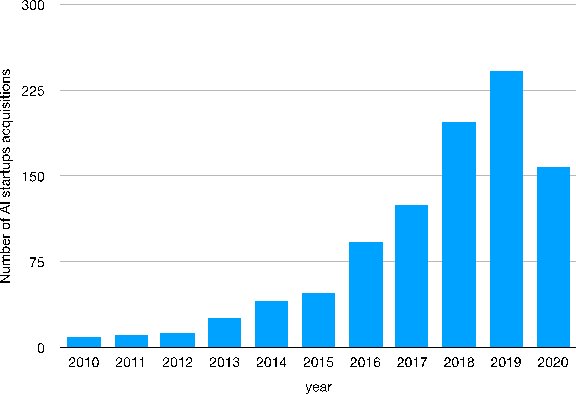
Worldwide number of AI startups acquisitions^42^ – 2010–2020 Source: Authors’ elaboration on Statista data

Figure 10 depicts the leading role of IBM in the AI applications market - with a global market share of 11.4%. Nevertheless, the largest share of applications is referred to ‘other firms’, reflecting an intense competitive dynamic. This may be related to the diffusion of AI into new areas and sectors (e.g., health, security) as well as to its already mentioned scalable nature. A rather different picture emerges if we concentrate our attention to acquisitions by key AI players, however. Figures 11–13 show the number of company (Fig. 11) and start-up (Fig. 12) acquisitions, including information on the key buyers (Fig. 13). Between 1997 and 2017, WIPO (2019) documents how the number of acquisitions increases exponentially: acquisitions grew on average by 5% between 2000 and 2012 and then strikingly accelerated with an average growth of 33% between 2012 and 2017. This seems to confirm that in the AI domain, appropriability opportunities are significantly among the top players.

Similar patterns can be detected by looking at start-up acquisitions. Figure 12 shows that the number of acquisitions of AI start-ups rises from 48 to 2015 to 242 acquisitions in 2019. In 2020, we record a decrease in the number of acquisitions due to the Covid-19 pandemic and the consequent socio-economic crisis.

As extensively discussed by WIPO (2019), leading AI companies are both consolidated IT incumbents - such as Apple, Microsoft, IBM and Intel - and relatively younger Big Tech companies - such as Alphabet/Google (accounting for 4% of the overall acquisitions), Amazon or Facebook.

This evidence provides three key messages: (i) the trend towards increasing market concentration continued to consolidate after 2016; (ii) massive appropriation of AI-related technological and market advantages by a few U.S.^43^ multinational companies is detected (Riakp and Lundvall, 2021); (iii) Alphabet (Google) outperforms other high-tech companies in terms of market acquisitions. Overall, strategic acquisitions emerge as a pivotal channel through which big-tech companies can conquer technological and market comparative advantages.

Once documented the degree of *indirect* appropriation of technological and market advantages related to AI-technologies *via* company acquisitions we can provide an assessment of the degree of *direct* appropriation *via* patent applications and ownerships.

To this end, we integrate our discussion with the detailed information provided by (WIPO, 2019). The Technology Trends Report discusses the huge leap experienced by the AI-related patents. Indeed, nearly 340,000 patent families and more than 1.6 million scientific publications related to AI have been registered and published between 1960 and 2018, and the number of AI-related annual patent registrations has been rapidly growing over the last ten years. WIPO (2019) identifies the top AI technologies, their functional applications and the fields of application. In addition, the Report allows mapping the distribution of patents among key companies and countries for each technology, application category and application field.

In Table 2 we summarize the main WIPO findings, lending support to the stylized history provided in Sect. 3. ML is indeed the dominant technological category within the AI domain, representing 89% of AI patent families, followed by Logic Programming and Fuzzy Logic. On the other hand, Computer Vision is the top AI functional application, representing 49% of the related patent families, followed by Speech Processing (14%) and NLP (13%), while Telecommunications (24%) and Transportations (24%) are the two top AI application fields in the AI patent families, with more than 50,000 patent filings each, followed by Life and Medical Sciences (19%).

**Table 2:** Top technology categories, application categories and application fields, and the related leading companies, by AI patents

Technology categories	Leading Companies	Application categories	Leading Companies	Leading Countries	Application fields	Leading Companies
Machine Learning	IBM - Microsoft	Computer Vision	Toshiba - Samsung	US - China	Telecommunications	Microsoft - Samsung
Logic Programming	IBM - Siemens	Speech Processing	Nuance Communications - Panasonic	US - Japan	Transportations	Toyota - Bosch
Fuzzy logic	Omron - Siemens	Natural Language Processing	IBM - Microsoft	US - China	Life and Medical Sciences	Siemens - Phillips

Source: WIPO Report 2019

When we look at to the key players, IBM and Microsoft maintain leading positions - with portfolios of, respectively, 8,920 and 5,950 total AI patents - especially in ML technologies - and also in a large number of ML subcategories, such as Probabilistic Graphical models, Rule Learning or Reinforcement Learning (IBM), Supervised Learning techniques (Alphabet), and Neural Networks (Siemens).

The picture slightly changes when it comes to identifying the top patent applicants related to AI functional applications. IBM and Microsoft confirm their leadership in NPL and Knowledge Representation and Reasoning, while Toshiba and Samsung dominate in Computer Vision, and Nuance Communications and Panasonic are the top applicants in Speech Processing.

Therefore, the descriptive evidence on AI’s development and adoption corroborate our qualitative discussion on technological discontinuities and application fields orienting the evolutionary trajectories of this technological domain.

Indeed, the convergence between ML – especially DL and ANNs - and Big Data exploitation techniques have represented a crucial trigger allowing AI to turn from a scientific niche to a rising industry with crucial commercial applications and the potential of being the GPP of the next future. Moreover, the top application categories in terms of AI-related patents refer to computer vision, speech recognition, and NLP, that is the most promising functional applications of AI in which Big Tech companies have been continuously investing.

Finally, the descriptive investigation of market-related factors reveals how the narrative describing the AI as characterized by a strong market dynamism - in terms of both technological and business opportunities for new entrants and start-ups – is misleading. What we find is a concentration of technological and market opportunities in the hands of traditional incumbents – such as Microsoft and IBM – and relatively younger digital giants – such as Google, Amazon or Facebook.

## Conclusions

This work provides a stylized history of AI discussing its implications for business model, organization and work. Adopting an *history-friendly* perspective, we identify technological, industrial, organizational *discontinuities* contributing to its growth and diffusion.

AI emerges as a complex technological domain shaped by *incremental* innovations. The latter are located at the intersection of different trajectories, embedded in the ICT *techno-economic paradigm.* From an evolutionary perspective, three different trajectories can be identified: (i) developments in statistical and computational theory and specific algorithmic techniques; (ii) data availability, closely linked to the establishment of the Internet; (iii) improvements in computational power and data storage capacity. These trajectories are punctuated by advances, in terms of knowledge, techniques and applications. Concerning its stylized history, we document how for a long time AI remained confined to a purely scientific-academic sphere with few or none industrial and business applications. A first set of crucial discontinuities regards the diffusion of microprocessors, digital devices, connected computers and then the Internet (Bonaccorsi and Moggi, 2019). Since then, AI becomes a key technology for corporations building their business model on data harvesting, e.g., Alphabet, Alibaba, Amazon. Indeed, AI technologies are at the center of strategies put forth by data-intensive corporation to control and eventually subordinate customers, suppliers and competitors. By the same token, AI allows to push forward the ‘lean-production logic’ (Dosi & Virgillito, 2019) increasing fragmentation, control, and exploitation of labor, in both manufacturing and services. AI is also linked to the spread of a new form of precarious work (e.g., ‘micro-workers’) mostly employed to ‘train and fix’ the algorithms underlying AI technologies. This is mirrored by a huge concentration of economic and technological power, as shown by the accumulation of patents in the hands of few data intensive corporations (Rikap & Lundvall, 2021). The diffusion of AI is thus contributing to the increase in the concentration of corporate power. By exploiting their technological comparative advantage and acquiring the most promising innovative start-ups, Big Tech companies tend to consolidate their dominant positions influencing both technological and market dynamics.

The diffusion of AI technologies is changing the nature of economic relationships both inside and outside the firm. As organizations become more flexible and fragmented, ‘central nodes’ become more powerful in ‘orchestrating’ value chains, markets and innovation ecosystems. Inside the firm, AI magnifies managerial skills, particularly concerning monitoring activities and real-time organizational adjustment in the face of external stimuli. Outside the firm, AI increases the power of firms that govern production chains by maximizing their ability to subordinate the actions of other nodes to their strategies. These developments are directly related to the scalar and modular nature of AI technologies as well as to the pervasiveness of their network and lock-in effects. As a result, the key discontinuities highlighted in this paper are the basis for a promising and articulated research agenda that will affect both economic and management studies in the near future. The changing nature of the AI technological domain and the lack of sound empirical evidence regarding its socio-economic impact make research in these areas particularly urgent.

## Appendix

**Table 1:** A time line of the history of AI

Year	General Description	Theoretical and algorithmic developments	Data availability	Computational power and data storage
XIV-XIX sec.	Theoretical roots of computational and probabilistic thinking			
1308		R. Lullo “Ars Magna”, theorization of logical machine		
1666		Leibniz “Dissertatio De Ars Combinatoria”		
1805		Legendre’s Least Square method		
1812		Laplace works on Bayes’ contributions formalizing the Bayes theorem		
1913		A. Markov introduces the Markov Chains		
1936	The rising of AI	Turing Machine to solve Hilbert’s ‘Entscheidungsproblem’		
1940–1950	First developments of ML algorithms and ANNs			
1943		Threashold Logic Unit (TLU), a formal design for Turing-complete artificial neurons		
1951		D. Edmons and M. Minsky, first neural network machine: Stochastic Neural Analog Reinforcement Calculator (SNARC)		
1951		A. Samuel (IBM), first machine playing checkers		
1956	AI as a proper research field	J. McCarthy coins the term ‘AI’ during the seminal workshop at Dartmouth College		
1956		A. Newell and H. Simon implement Logic Theorist (LT)		
1958		F. Rosenblatt working at the Cornell Aeronautical Laboratory introduces his ANN: the perceptron		
1960s	Extensive application of probabilistic methods and developments of ML algorithms			C. Bachman designs the Integrated Database System (IDS), the first Database Management System (DBMS)
1963		D. Michie implements a machine able to play Tic-Tac-Toe via Reinforcement Learning (RL)		
1965				Moore’s Law on exponential growth of the chip power. The number of transistors in a dense Integrated Circuit (IC) is expected to double every two years
1967		Nearest Neighbor algorithm as a first step towards pattern recognition		
1970s	The ‘AI winter’	Limitations of ML applications and developments highlighted by Minsky and Papert’s book “Perceptrons” (1969)		
1971				Bachman’s Database Task Group presents the standard language Common Business Oriented Language (CBOL)
1973				M. Stonebraker and E. Wong (UC Berkley) start the Interactive Graphics and Retrieval System (INGRES) project on relational database systems using the query language QUEL
1974				IBM develops the Structured Query Language (SQL)
1979		K. Fukushita work on the neocognitron ANN laying the groundwork for Convolutional Neural Networks (CNNs)		
1980s	Revival of ML research projects and first commercial AI products	Diffusion of Expert Systems within US industries		Complementary Metal-Oxide-Semiconductors (CMOS) technology, as a Development of Metal-Oxide-Semiconductors (MOS) Very Large-Scale Integration (VLS): practical development of ANNs
1982		J. Hopfield introduces Recurrent Neural Networks (RNNs) as Content-Addressable Memory (CAM) systems		
1986		D. Rumelhart, G. Hinton and R. Williams introduce the backpropagation technique to train ANNs		
1989		C. Watkins develops Q-learning by improving RL methods		
1989		Axcelis, Inc. (US) commercializes Evolver, the first software package for PC using genetic algorithms		
1989		Convolutional Neural Networks (CNNs) to shape and image recognition and classification		
1990–2000	Exploitation of ML-AI for a wide and increasing range of knowledge domains and application fields. From theory-driven to data-driven ML research and development			
1991			World Wide Web	
1992		G. Tesauro implements TD-Gammon, an ANN program playing Gammon		
1995		Introduction of random forest algorithms		
1995		C. Cortes and V. Vapnik first work on Support Vector Machines (SVMs), widely used to solve many Natural Language Processing (NLP) problems		
1997		S. Hochreiter and J. Schmidhuber introduce the Long Short-Term Memory (LSTM) Recurrent Neural Networks (RNNs)		
1997		IBM’s Deep Blue vs. chess champion G. Kasparov		
1998		S. Brin and L. Page (Stanford University) introduce PageRank algorithm	Release of the Modified National Institute of Standards and Technology (MNIST) Database to train image processing systems	
2000s			Web 2.0	Diffusion of Not only-Structured Query Language (NoSQL) and In-Memory Databases
2002				Amazon Web Services offers cloud-based storage and computing power to users
2004			Facebook	
2005			YouTube	
2007			Apple launches the first iPhone	
2009			ImageNet database	M. Zaharia (UC Berkley’s AMPLab) develops the Apache Spark open source framework to exploit and update Big Data
2008–2015			Web 3.0 - S*emantic Web*	
2010				Microsoft and Google launch their cloud, Microsoft Azur and Google Cloud Storage
2011		IBM’s Watson beats two human champions at *Jeopardy!* by using NLP ML and information retrieval techniques		
2012		Google Brain team creates an ANN capable of recognizing cats from unlabeled YouTube video frames		
2013		DeepMind implement a CNN able to play Atari via Deep Learning		
2014		Facebook researchers present DeepFace, a neural network system for face recognition		
2014		Google’s researchers present Sibyl, a ML-driven platform for users’ behavior prediction and recommendations		
2016		Google’s AlphaGo plays Go		
2017		Maluuba (Microsoft) implements a RL algorithm able to play and master Pacman game		

## Footnotes

1 AI refers to a broad and rapidly expanding field of technologies. As a result, the literature does not provide a single definition (Van Roy et al., 2020). The OECD proposes the following definition: “*An AI system is a machine-based system that can, for a given set of human-defined objectives, make predictions, recommendations or decisions influencing real or virtual environments. It uses machine and/or human-based inputs to perceive real and/or virtual environments; abstract such perceptions into models (in an automated manner e.g. with machine learning (ML) or manually); and use model inference to formulate options for information or action. AI systems are designed to operate with varying levels of autonomy*.” (Lane and Saint-Martin, 2021, p. 18). As for the economics domain, Acemoglu and Restrepo (2019, p.1) define AI as “*the study and development of intelligent (machine) agents, which are machines, software or algorithms that act intelligently by recognizing and responding to their environment*.”

2 The lean production model – i.e. *just-in-time* and *just-in-sequence* – is aimed at reducing costs in production, logistics and warehousing through the meticulous synchronisation of all production phases. In a context of increasing digitalization and fragmentation of production chains, AI together with other ‘4.0 technologies’ – i.e. Internet of Things (IoT) and Big Data – enhances synchronization and monitoring capabilities, making it possible to connect machines, workers and plants irrespective to their geographical localization.

3 Producing, developing and (even less so) adopting AI technologies is of course not sufficient to set a dominant data-intensive business model as those characterizing contemporary digital monopolies, e.g., Alphabet, Amazon, Alibaba, Meta. The power of such corporations is in fact driven by a complex array of factors beyond AI technologies. However, AI is crucial to exploit data, purse efficiency maximization strategies as well as to govern production, retail and innovation networks.

4 UNCTAD (2021) has recently documented how AI investments and patents are indeed dramatically concentrated among few corporations, mostly based in the US and China.

5 Some authors have recently addressed this question without, however, providing conclusive answers (see, among others, Trajtenberg, 2018; Varian, 2018; Cockburn et al. 2018; Iori et al., 2021).

6 Nuvolari (2019) and Cetrulo and Nuvolari (2019) argue that AI should be included within the broader ICT techno-economic paradigm emphasizing the incremental nature of such technological domain.

7 It should be mentioned that a number of authors have explicitly referred to AI as a GPT. The OECD (2019) refer to AI as a GPT due to its potential application across a broad variety of sectors and occupations, its incremental nature and complementarity with other technologies Agrawal et al., (2019) argue that AI can be qualified as a a GPT due to its ability to produce predictions. The latter, in turn, may be used as strategic input to support decision-making in a variety of sectors and occupations. Both Brynjolfsson et al., (2019) and Cockburn et al., (2019) emphasize the general-purpose nature of Machine Learning, a key component of AI, since it is considered not only to offer productivity gains across a wide variety of sectors, but also to offer transformation of the innovation processes within those sectors.

8 Machine Learning (ML) is a subfield of the AI domain involving all those techniques related to the implementation of algorithmic procedures enabling (intelligent) machines to *learn* and adapt to changing environments. ML has been developed along three main learning paradigms, that is: (i) Supervised Learning (SL); (ii) Non-Supervised Learning (NSL); Reinforcement Learning (RL). SL and NSL differ depending on the exploitation of, respectively, labeled/structured or unlabeled/unstructured datasets in order to *train* the algorithm. On the contrary, RL implement sequential decision and learning protocols assigning numeric values (rewards) whenever the algorithm undertake the correct decision. As each sequential decision step depends on the current state of the system, these learning protocols are crucially built on Markov Decision Process (MDP) techniques.

9 As we will discuss in Sect. 2.1, a further subfield of ML is represented by the *Deep Learning* (DL), that is a set of techniques allowing for the automatic detection of data representation and classification by the intelligent machine. Artificial Neural Networks (ANNs) are one of the tools available for DL procedures and represent computational models inspired to the functioning of biological neural networks to solve mathematical, statistical and engineering informational problems. Thus, an ANN model is populated by an adaptive network of interconnected nodes (i.e. the artificial neurons) exchanging internal and external information flows.

10 UNCTAD (2021) reports that global investment in AI companies has increased tremendously and persistently in the recent past. In 2019, privately held AI companies attracted nearly $40 billion in disclosed equity. True numbers could however be higher (as much as $74 billion) because some transactions do not have publicly disclosed values. Worldwide, the US has the world’s largest investment market in privately held AI companies.

11 As Rikap & Lundvall (2021) emphasize, the performance of AI algorithms is strongly dependent on data inflow. As larger and diverse is the amount of data as effective the learning process for the algorithms and machines relying on them.

12 It is worth noticing that a key role in the AI market is also played by historical players of the ICT sector such as, for example, IBM.

13 The machine was never constructed by Babbage, but it interestingly represented a source of inspiration for a certain literary imagery during the 1990s, as in the dystopic novel “*The Difference Engine*” (1990) by W. Gibson and B. Sterling.

14 In 1936, A. Turing in his “*On Computable Numbers, with an Application to the Entscheidungsproblem*” introduced an algorithmic computational model, based on his famous theoretical *machine*, capable of solving the ‘decision problem’ proposed by D. Hilbert (the *Entscheidungsproblem*) and laying the foundations of modern informatics and computational theory.

15 Such a slowdown of scientific and research activities related to AI is highlighted, in 1969, by Minsky and Papert in their book “Perceptrons”.

16 Recurrent (RNN) and Convolutional Neural Networks (CNNs) are, respectively, two different developments of neural networks techniques. In particular, RNNs models entail non-linear transformation node functions with cyclical (recurrent) inflow and outflow of information/values between upper and lower layers of the network, whereas CNNs are feed-forward models wherein the connectivity patterns among the artificial neurons mimic the function of biological visual cortexes. Therefore, the latter have been increasingly developed and implemented for specific functional applications of AI systems, such as image and video recognition or natural language processing.

17 As extensively discussed by Taddy (2019), we can refer to General Purpose ML as the currently unfolding version of ML techniques, mainly based on DL and DNN tools.

18 Long Short-Term Memory Recurrent Neural Network (LSTM-RNN) are RNNs models wherein each neuron has multiple *gates* enabling it to autonomously choosing during the training/learning process which part of the information has to be memorized or not.

19 As Rikap & Lundvall (2021) highlight, the linkage between ML algorithms and Big Data is key to the diffusion and the growing importance of AI, particularly for large Internet corporations. Algorithms are at the basis of ML models and their performance, i.e. ability to learn, is directly related to the amount and quality of data algorithms can rely on. As larger the amount of inflowing data as higher the potential to increase AI’s performance. As a result, corporation controlling large data networks are in the position of dominating the development of key AI technologies.

20 In their ‘The De-Democratization of AI: Deep Learning and the Compute Divide in Artificial Intelligence Research’, Ahmed & Wahed (2020) document that, between 2000 and 2019, large high-tech corporations have increased their participation in major AI conferences. Looking at the share of papers whose authors are affiliated with private corporation, the authors display a strong increasing trend.

21 Similar evidence is provided by Castro et al., (2019)

22 Relying on WIPO (2019)’s AI patents mapping, Rikap & Lundvall (2021) distinguish between functional and industrial AI applications. Key applications are: knowledge representation and reasoning, planning and scheduling, control methods, computer vision, speech processing, predictive analytics, distributed AI, robotics and natural language processing. Concerning industrial applications, the three top industries are: transport, telecommunications and life and medical science.

23 IMDBs are Database Memory Systems (DBMS) relying on main memory – instead of hard disks as for traditional DBMSs – for computer data storage.

24 Instead of using tabular relations as in Relational (traditional) Database Management Systems (RDBMS), NoSQL – as the name specifies – may either use structured (SQL) or unstructured query languages in order to deal with Big Data and real time web applications.

25 For a description of how Maluuba exploited RL and HRA to trainn an AI system able to in play and master the Pac-Man game see https://blogs.microsoft.com/ai/divide-conquer-microsoft-researchers-used-ai-master-ms-pac-man/.

26 Similar arguments, pointing to the fast improvements in ML-based AI systems, are those put forth by Brynjolfsson and Mitchell (2017), Brynjolfsson et al., (2018) and Webb (2020).

27 The name refers to the (fake) chess-player automata invented by W. Von Kempelen as a homage to Maria Theresia von Österreich in 1769. The ‘Turk’ was, in fact, operated by a hidden human chess player.

28 The Amazon MTurk’s home page reads: “[…] *MTurk enables companies to harness the* collective intelligence, *skills, and insights from a global workforce to streamline business processes, augment data collection and analysis, and accelerate machine learning development.*” The reference to the *collective intelligence* may remind us of the well-known “*Fragment on Machines*” contained in K. Marx’s *Grundrisse* (1857-58), one of the first brilliant prefiguration of the ‘information society’. However, far from envisaging the development of *collective intelligence* in a Marxian perspective, the presentation provided by Amazon MTurk itself, provides some points of reflection on the critical current state of human-machine interaction.

29 As discussed by Rikap & Lundvall (2021), analogous labor platforms currently operate in China.

30 In 2013 Amazon bought the Polish-based Ivona specialized in voice recognition technologies to compete against Apple’s Siri (for an analysis of Amazon’s acquisitions, see Coveri et al., 2021).

31 For further details see https://www.statista.com/.

32 Data are collected by Statista from Venture Scanner.

33 Data are collected by Statista from Worldwide Business Research (WBR). RPA is defined as a software development toolkit allowing non-specialized operators to implement software robots (the so-called *bots*) in order to automate rules-driven business operations and processes. IPA refers to the usage of AI technologies to automate a business function or a specific segment of the workflow.

34 Data are collected by Statista from WBR and International Data Corporation (IDC).

35 Data are collected by Statista from 451 Research’s survey Voice of the Enterprise. The survey collect data from 106 respondents operating in the worldwide retail industry.

36 The selected countries are: Australia, France, Germany, India, Italy, Netherlands, Spain, Sweden, United Kingdom, United States.

37 Data are collected by Statista from Capgemini. The survey provides information on 850 respondents among senior IT executives from selected countries: Australia, France, Germany, India, Italy, Netherlands, Spain, Sweden, United Kingdom, United States.

38 Data are collected by Statista from McKinsey’s 2019 online survey collecting information from 2.935 respondents representing the full range of regions, industries, companies size, functional specialties, and tenures.

39 Data are collected by Statista from three different source, as follows: (i) IDC for what concerns AI systems related to hardware, software and services; (ii) Tractica on B2B AI-related software covering vision, language and analytics categories; (iii) Grand View Research (GVR) fo data on AI segments including hardware, software and services, and AI functional applications including DL, ML, natural language processing and machine vision.

40 Data are collected by Statista from McKinsey’s 2019 online survey collecting information from 2.935 respondents representing the full range of regions, industries, companies size, functional specialties, and tenures.

41 Data are collected by Statista from IBM and IDC.

42 Data are collected by Statista from CB Insights.

43 Even though, as recently discussed by Rykap and Lundvall (2021) Chinese tech-giants such as Alibaba and Tencent are rapidly getting a leadership position in the banking and finance AI application fields.
